# Lithium and its effects: does dose matter?

**DOI:** 10.1186/s40345-024-00345-8

**Published:** 2024-06-24

**Authors:** Mirko Manchia, Pasquale Paribello, Martina Pinna, Luca Steardo, Bernardo Carpiniello, Federica Pinna, Claudia Pisanu, Alessio Squassina, Tomas Hajek

**Affiliations:** 1 Section of Psychiatry, Department of Medical Sciences and Public Health, University of Cagliari, Cagliari, Italy; 2 Unit of Clinical Psychiatry, University Hospital Agency of Cagliari, Cagliari, Italy; 3 Department of Pharmacology, Dalhousie University, Halifax, Nova Scotia, Canada Italy; 4 Unit of Forensic Psychiatry, Health Agency of Cagliari, Cagliari, Italy; 5 Psychiatry Unit, Department of Health Sciences, University of Catanzaro Magna Graecia, Catanzaro, Italy; 6 Section of Neuroscience and Clinical Pharmacology, Department of Biomedical Sciences, University of Cagliari, Cagliari, Italy; 7 Department of Psychiatry, Dalhousie University, Halifax, NS Canada

**Keywords:** Micro-dose, Suicide, GSK-3Beta, BDNF, Drinking water

## Abstract

**Background:**

Decades of clinical research have demonstrated the efficacy of lithium in treating acute episodes (both manic and depressive), as well as in preventing recurrences of bipolar disorder (BD). Specific to lithium is its antisuicidal effect, which appears to extend beyond its mood-stabilizing properties. Lithium’s clinical effectiveness is, to some extent, counterbalanced by its safety and tolerability profile. Indeed, monitoring of lithium levels is required by its narrow therapeutic index. There is consensus that adequate serum levels should be above 0.6 mEq/L to achieve clinical effectiveness. However, few data support the choice of this threshold, and increasing evidence suggests that lithium might have clinical and molecular effects at much lower concentrations.

**Content:**

This narrative review is aimed at: (1) reviewing and critically interpreting the clinical evidence supporting the use of the 0.6 mEq/L threshold, (2) reporting a narrative synthesis of the evidence supporting the notion that lithium might be effective in much lower doses. Among these are epidemiological studies of lithium in water, evidence on the antisuicidal, anti-aggressive, and neuroprotective effects, including efficacy in preventing cognitive impairment progression, Alzheimer’s disease (AD), and amyotrophic lateral sclerosis (ALS), of lithium; and (3) revieweing biological data supporting clinically viable uses of lithium at low levels with the delineation of a mechanistic hypothesis surrounding its purported mechanism of action. The study selection was based on the authors’ preference, reflecting the varied and extensive expertise on the review subject, further enriched with an extensive pearl-growing strategy for relevant reviews and book sections.

**Conclusions:**

Clinical and molecular effects of lithium are numerous, and its effects also appear to have a certain degree of specificity related to the dose administered. In sum, the clinical effects of lithium are maximal for mood stabilisation at concentrations higher than 0.6 mEq/l. However, lower levels may be sufficient for preventing depressive recurrences in older populations of patients, and microdoses could be effective in decreasing suicide risk, especially in patients with BD. Conversely, lithium’s ability to counteract cognitive decline appears to be exerted at subtherapeutic doses, possibly corresponding to its molecular neuroprotective effects. Indeed, lithium may reduce inflammation and induce neuroprotection even at doses several folds lower than those commonly used in clinical settings. Nevertheless, findings surrounding its purported mechanism of action are missing, and more research is needed to investigate the molecular targets of low-dose lithium adequately.

## Introduction

Decades of clinical research have demonstrated the efficacy of lithium in treating acute episodes (both manic and depressive), as well as in preventing recurrences of bipolar disorder (BD) (Alda 2015; Licht 2012; Severus et al. 2018). Specific to lithium is its antisuicidal effect (Cipriani et al. 2013; Smith and Cipriani 2017; Tondo and Baldessarini 2018), which appears to extend beyond its mood-stabilizing properties (Ahrens and Müller-Oerlinghausen 2001; Manchia et al. 2013). Lithium’s clinical effectiveness is, to some extent, counterbalanced by its safety and tolerability profile (McKnight et al. 2012). Indeed, lithium treatment is associated with an increased risk of reduced urinary concentrating ability, hypothyroidism, hyperparathyroidism, and weight gain (McKnight et al. 2012). In this context, the decision to start BD patients on lithium must be based on a careful assessment of the risks and benefits associated with the treatment.

Thus, lithium haematic concentrations need to be regularly monitored to address possible adherence issues, which may be particularly frequent during therapy (Baldessarini and Tondo 2022), as well as to avoid reaching toxic levels (De-Paula et al. 2016). Past reports suggest that episodes of lithium haematic concentrations exceeding the 0.8 mEq/L threshold may be more damaging in terms of nephrotoxicity as compared with other factors such as the overall duration of therapy or average lithium levels (De-Paula et al. 2016). Further, the impact of treatment discontinuation in individuals on long-term lithium therapy is particularly relevant (Kumar et al. 2023). Therefore, it is considered of utmost importance to try to address risk factors and manage conditions that might otherwise limit or contraindicate lithium therapy. These elements rekindled the interest of the medical community in exploring the value of lower lithium concentrations compared to the more established threshold of 0.6 mEq/l for the treatment of mental disorders and, more specifically, BD (Taylor et al. 2021). A precise threshold for therapeutic efficacy of hematic lithium concentrations would also vary depending on numerous elements, not least its indications. Certain lines of evidence suggest that for prevention of depressive episodes, a level ≥ 0.4 mEq/l may be required. However, this level might not be sufficient to prevent manic episodes (Nolen et al. 2019; Taylor et al. 2021). On the other hand, the evidence for acute treatment of BD depressive episodes is derived mainly from methodologically weak studies and is not particularly robust even when considering regular dosing (Meyer and Stahl 2023). Therefore, it might also be sensible not to expect significant effects even with subtherapeutic dosing in the absence of further evidence of the opposite. Findings on unipolar depression seem to indicate that lithium augmentation of antidepressant therapy may represent a valuable option, especially considering a relatively more favourable side effect profile as compared with second-generation antipsychotic augmentation (Meyer and Stahl 2023; Strawbridge et al. 2023). Here, we considered studies reporting on subtherapeutic doses of lithium when the specified range of lithium concentrations comprises blood lithium concentrations below the 0.4 mEq/l threshold. Certain lines of evidence suggest that, especially in the context of unipolar depression therapy, the minimum effective dose could be as low as 0.4 mEq/L (Ercis et al. 2023; Taylor et al. 2021), with possible improvements in terms of tolerability as compared with the most established target range comprised between 0.6 and 0.8 mEq/L, but at the cost of being potentially less effective as a prophylactic intervention (Taylor et al. 2021).

Once started on lithium, patients with BD need accurate longitudinal clinical monitoring of adverse effects, which should be often performed on a very long-term, if not lifelong, basis (Alda and Manchia 2018). Ideally, clinical monitoring should be performed in specialised units, termed “Lithium clinics”, which are not confined to the assessment of adequate (therapeutic) serum lithium levels but often propose complementary approaches such as psychoeducation, rehabilitation, cognitive therapy, social rhythm therapy, and employment counselling (Osher et al. 2010). This approach has been shown to have beneficial effects in ensuring adequate treatment adherence and, in general, in improving clinical outcomes (Licht et al. 2001; Masterton et al. 1988).

Monitoring of lithium levels is required by its narrow therapeutic index. As previously reported, there is a general consensus that adequate serum levels should be above 0.6 mEq/L to achieve clinical effectiveness, while toxicity might present at levels of 1.0 mEq/L and higher (Meyer 2017). However, discrepant findings on the appropriateness of this threshold to obtain clinical efficacy have been present since early clinical studies. For instance, Schou indicated that although levels of 0.6 mEq/L might be adequate in some patients, levels of at least 0.8 mEq/L might be needed to ensure clinical effectiveness (Schou 1968). On the other hand, Zall et al. indicated that a range between 0.3 mEq/L and 0.6 mEq/L was more than adequate for clinical efficacy (Zall et al. 1968).

In general, although the validity (and the clinical significance) of the 0.6 threshold has been largely undisputed, its implementation has been based on relatively few studies with significant methodological shortcomings. A recent systematic review and recommendation from the ISBD/IGSLI concluded that in the absence of more rigorous evidence, the 0.6 mEq/l threshold was based on a study selection derived from lowering the standard for selection criteria (Nolen et al. 2019). Only seven studies were ultimately identified, and none was considered of moderate or high quality according to the JADAD quality scale for randomised clinical trials (Nolen et al. 2019). More specifically, only three out of seven studies were described as double-blind, but only one of them adequately described the blinding process itself. Significantly, only four studies adequately described the reason for dropouts, limiting the interpretability of the overall findings (Nolen et al. 2019). Furthermore, the 0.6 mEq/l threshold has been automatically extended to other pharmacodynamic properties of lithium, such as the antisuicidal or neuroprotective effects, for which it has not been systematically tested. And it is not secondary that clinical and basic research also relies on these levels. Evidence of reduced hospitalisation rates and possible suicide preventive effects of lithium exposure from drinking water have sparked interest in exploring the potential role of subtherapeutic blood concentrations and lithium microdoses (Eyre-Watt et al. 2021). Lithium microdoses are defined based on daily doses, which are considered well below the level known to exert therapeutic benefit for formally approved indications of lithium salts. A precise and shared definition for micro dosing is still lacking, but doses equal to 300 µg daily have been described and tested in the past in a randomized clinical trial (RCT) aiming at testing its potential worth in influencing cognitive impairment progression in Alzheimer’s disease patients (Andrade Nunes et al. 2013). The mere definition of therapeutic doses of lithium may also lead to possible confusion as it would not necessarily be specifically linked to a precise peripheral blood concentration range across different subjects and even in the same subject with physiological or pathological changes, potentially representing a weakness for this specific definition. Relevant to the discussion of subtherapeutic and alternative sources of lithium exposure, several different forms of lithium supplement exist, such as lithium orotate salts. These products are available for sale on the internet in several countries, albeit no clear evidence surrounding their safety or usefulness for neuropsychiatric illnesses exists to this day (Meyer and Stahl 2023). Still, this might represent a relatively growing concern for the public and clinicians alike as it may still represent a relatively significant source of lithium exposure.

In this context, our review has a threefold aim: (1) to review and critically interpret the clinical evidence supporting the use of the 0.6 mEq/L threshold; (2) to report a narrative synthesis of the evidence supporting the notion that lithium might be effective in much lower doses. Among these are epidemiological studies of lithium in water, evidence on the antisuicidal, antiaggressive, and neuroprotective effects, including efficacy in cognitive impairment, Alzheimer’s disease (AD), and amyotrophic lateral sclerosis (ALS), of lithium; (3) to review biological data supporting the action of lithium at low levels with the delineation of a mechanistic hypothesis (to which targets does lithium might have sufficient affinity to affect them at even very low doses? ).

## Methods

This study was designed as a narrative review of the available literature in the field with a selection of papers based on criteria of relevance determined by the authors. The selection was further enhanced by a literature review using the following search terms on PubMed/Medline: “lithium” AND (“levels” OR “threshold”) AND “serum” AND “bipolar disorder”. Similarly, searches were performed using the terms: “lithium” AND (“levels” OR “threshold”) AND “biological effects”; “lithium” AND (“levels” OR “threshold”) AND (“MCI” OR “mild cognitive impairment”); “lithium” AND (“levels” OR “threshold”) AND “amyotrophic lateral sclerosis”. The search did not have a time limit or exclusion criteria. An extensive pearl-growing strategy was applied to pertinent reviews, books, and other sources to enlarge the scope of our search further.

### Lithium therapeutic serum levels: review of the clinical evidence

RCTs and observational studies in mood disorders.

#### Evidence for maintenance treatment in bipolar disorder

The seminal double-blind discontinuation study of Baastrup et al. showed unequivocally the efficacy of lithium in preventing recurrences in BD and unipolar depressed subjects (Baastrup et al. 1970). Lithium-treated patients had serum levels between 0.6 and 1.5 mEq/L (Baastrup et al. 1970). This was also confirmed by the work of Hullin et al. (1972). Further, the RCT of Gelenberg et al. showed that patients treated with standard levels of lithium (0.8-1.0, median 0.83 mEq/l) had a significantly decreased risk of relapse compared to those at low levels (0.4–0.6, median 0.54 mEq/l) (Gelenberg et al. 1989). In addition, this effect appeared to extend also to subsyndromal mood symptoms (Keller et al. 1992). Interestingly, the same group showed that standard levels (0.8-1.0 mEq/l) of lithium were associated with higher psychosocial functioning compared to low levels beyond the effect determined by the reduction of illness activity (Solomon et al. 1996). One interesting hypothesis was formulated concerning the presence of specificity in the prophylactic efficacy of lithium for the diverse mood phases of BD, depending on its different levels. Indeed, a review of clinical trials of lithium for maintenance [divided into trials at low (below 0.6 mEq/l), medium (0.6 to 0.8 mEq/l) and high (above 0.8 mEq/l) serum levels] showed that the percentage of depressive recurrences in the groups with low, medium and high lithium levels differed in a clinically and statistically significant manner (12% vs. 38% vs. 64%, *p* < 0.0001), suggesting that low lithium levels might be effective in preventing depression. In contrast, higher blood levels are needed to prevent hypo-manic or mixed states (Kleindienst et al. 2005). A subsequent multicenter clinical trial showed that average lithium levels preceding the reappearance of manic or mixed symptomatology were lower than levels preceding the reappearance of depressive symptoms (0.53 ± 0.13 vs. 0.66 ± 0.21 mEq/l, *p* = 0.01), suggesting that manic or mixed recurrences might rather occur lower lithium levels, whereas the depressive pole prevails in the higher range (Kleindienst et al. 2007). Even when accounting for the index episode in a logistic model, the presence of (hypo)-manic index episode was not significantly associated with the polarity (Kleindienst et al. 2007). A series of trials showed that lithium at low doses might be effective in preventing depressive recurrences but not hypomanic/manic (Severus et al. 2010; Severus et al. 2009), although this evidence was countered by the findings of Nolen et al. showing that lower lithium levels were less effective in preventing recurrences of both polarities (Nolen and Weisler 2013). Importantly, Nolen et al. indicated that the evidence for the identification of an optimal serum lithium threshold remains scant and that levels should be tailored to the specific population considered, i.e. in the elderly, lower levels, usually 0.40-0.60 mEq/l, could exert a therapeutic effect maintaining an adequate safety profile (Nolen et al. 2019). Although data remain inconclusive, there is consensus that lithium exerts maximal mood stabilising properties at doses corresponding to serum concentrations of 0.6 and higher, possibly more specifically for hypo/manic recurrences. Other clinical effects might result from its use at much lower doses, as discussed in the following sections.

#### Evidence for acute mania treatment

A series of studies compared the efficacy of lithium versus chlorpromazine in the treatment of manic symptoms using a double-blind, randomised design (Johnson et al. 1971; Platman 1970; Shopsin et al. 1975; Spring et al. 1970; Takahashi et al. 1975). These studies found that lithium was superior to chlorpromazine in treating mania, but there was substantial heterogeneity in lithium levels reached to obtain this clinical effect. For instance, Spring et al. showed a higher, although not statistically significant difference, improvement in manic symptoms under lithium compared to chlorpromazine with lithium levels in the range between 0.6 and 1.3 mEq/L (Spring et al. 1970). This was similar to levels achieved by Johnson et al. (1971) and Shopsin et al. (1975), but in contrast with the findings of Takahashi et al., who found that the mean serum levels needed to achieve response was around 0.50 mEq/L (Takahashi et al. 1975). Another randomised alternating-dose, double-blind, crossover design trial showed that lithium was superior to placebo in controlling manic symptoms; however, there was no correlation between serum lithium levels and clinical response to lithium (Stokes et al. 1971). Further, the same group used the same study design with high (0.72 mEq/kg/day) and medium (0.50 mEq/kg/day) lithium chloride doses, showing that both were more effective than placebo in controlling manic symptoms (Stokes et al. 1976). Importantly, there was no higher statistically significant clinical efficacy of the high dose compared to the medium dose of lithium (Stokes et al. 1976). Similar findings show the superiority of lithium versus placebo, but a series of studies showed a lack of correlation between mean serum lithium levels and clinical response (Coppen et al. 1971; Prien et al. 1972). Of interest is that the trial of Cundall et al. did not find an association between low serum lithium levels and lack of clinical response (Cundall et al. 1972). This finding was in line with the study of Prien and Caffey, which showed that treatment response was achieved with higher, although not significantly different from those of non-responders, mean serum lithium levels (0.8 mEq/l in responders versus 0.63 mEq/l in non-responders) (Prien and Caffey 1976).

### Lithium evidence of efficacy outside of the bipolar disorder: effectiveness of subtherapeutic levels

#### Lithium in drinking water and neuropsychiatric outcomes

The first report suggesting an association between a lessened number of psychiatric hospitalisations and lithium concentrations in drinking water dates back to 1970 (Dawson et al. 1970). Subsequently, a study by Schrauzer and Shrestha (1990) showed that lithium levels in drinking water were negatively correlated with suicide incidence rates. The range of concentrations associated with this protective effect (70–170 µg/L) was far below the therapeutic dosage used in the management of BD (Schrauzer and Shrestha 1990).

However, this field of research has been particularly controversial considering that even in areas with exceptionally high concentrations of lithium in drinking water, the resulting exposure would still be far lower than the one thought to be relevant for the treatment of psychiatric disorders (Araya et al. 2022; Harari et al. 2015). Even so, this exposure appears to maintain the potential to exert noticeable physiological effects (Harari et al. 2015). A growing body of evidence suggests that exposure to sub-therapeutic lithium concentration through drinking water may impact a variety of clinically significant outcomes for the neuropsychiatric field, such as reducing dementia risk (Duthie et al. 2023; Parker et al. 2018), suicide risk (Barjasteh-Askari et al. 2020), homicide risk (Giotakos et al. 2015), psychiatric hospital admissions (Eyre-Watt et al. 2021), but potentially increase autism spectrum disorder risk in prenatal exposure (Liew et al. 2023). These data were further and robustly supported by a recent meta-analysis of 9 studies (Memon et al. 2020), which suggested that naturally occurring lithium in drinking water may reduce the risk of suicide and possibly help in mood stabilisation.

Overall, the interpretation of the available evidence appears particularly complex considering the difficulty in addressing possible confounders influencing the outcomes (e.g., socioeconomic factors on suicides), heterogeneity in the reported outcomes, sex differences in the observed associations, and the unfeasibility of assessing the amount of lithium exposure through routes other than drinking water (e.g., food) (Duthie et al. 2023). Critical for the field is to define the impact of prenatal exposure to lithium in drinking water on fetal development, as past reports have suggested the potential for some health benefits, albeit non-unanimously, but also for possible detrimental effects (Harari et al. 2015) even at comparatively similar concentrations (Eyre-Watt et al. 2021; Liew et al. 2023). Indeed, several papers investigated the possible worth of lithium in drinking water in preventing dementia, finding either no benefits or mixed evidence for a protective effect for concentrations higher than 30 µg/L, but not for low-mid level exposure. Interestingly, mid to low-level exposure has been reported to be associated with an increased dementia risk as compared with the lower decile of lithium concentration (Duthie et al. 2023; Kessing et al. 2017). In addition, the study by Fajardo and coworkers showed that trace lithium in drinking water in Texas was negatively linked not only with changes in AD mortality but also with obesity and type 2 diabetes, which are important risk factors for AD (Fajardo et al. 2018). A 2021 meta-analysis condensing the results of 14 studies, reporting on data originating from 2678 regions and containing 113 million people, concluded that higher concentrations of lithium in drinking water exposure were associated with reduced psychiatric hospitalisation and suicide rates (Eyre-Watt et al. 2021). Early enthusiasm for the evidence of potential benefits surrounding lithium in drinking water exposure has led some to ponder the possibility of supplementing lithium as a public health measure in areas with low lithium concentrations. However, the currently unaddressed gaps in our knowledge of lithium in drinking water effects on suicide and psychiatric hospitalisation (where no dose-relationship has been established) (Eyre-Watt et al. 2021), the unclear effects related to prenatal exposure (Harari et al. 2015; Liew et al. 2023), the non-linear association between lithium in drinking water concentrations and dementia risk and the inconsistency of results when sex stratification is considered (Duthie et al. 2023), suggest caution in the interpretation of current data (Luca and Luca 2022). Indeed, the ongoing debate surrounding prenatal exposure to fluorinated water and lower IQ (Green et al. 2019) should represent a cautionary tale, further underscoring how even well-consolidated practices in public health may require continuos scrutiny to better grasp their impact on the general public especially in a lifespan perspective.

#### Lithium in neurocognition and ALS

The data on lithium in drinking water prompted several clinical investigations on the efficacy of lithium microdoses in restoring cognitive impairment in AD. These investigations were based on the hypothesis that even subtherapeutic doses of lithium could interfere with pathways involved in neuroprotection. Some RCTs explored the efficacy of sub-therapeutic doses of lithium in mild cognitive impairment (Forlenza et al. 2011, 2019). In the study, during a 12-month, double-blind trial in 45 patients with amnestic mild cognitive impairment, lithium (0.25–0.5 mEq/l) was superior to placebo in decreasing CSF concentrations of P-tau and increased the performance on the cognitive subscale of the Alzheimer’s Disease Assessment Scale and in attention tasks (Forlenza et al. 2011). The second RCT was performed in a larger sample (*n* = 61) of older adults with MCI with a much longer duration: 2 years of the double-blind phase, followed by an additional 24 months in the single-blinded phase (Forlenza et al. 2019). Lithium treatment was associated with better performance on memory and attention tests after 24 months and with a significant increase in CSF amyloid-beta peptide (Aβ1–42) after 36 months (Forlenza et al. 2019). Another clinical trial explored the efficacy of low-dose lithium in treating behavioural symptoms in AD, showing improvement in the global level of function and excellent safety (Devanand et al. 2022), although agitation was not affected. The findings on the effect of low-dose lithium on neurocognition in AD are of great relevance. A recent systematic review and meta-analysis showed that low-dose lithium significantly outperformed donanemab, aducanumab and placebo at the assessment with the Mini-Mental State Examination (Terao and Kodama 2024). In addition, low-dose lithium appeared to be safer and better tolerated than aducanumab, lecanemab and donanemab (Terao and Kodama 2024). The higher efficacy of low-dose lithium compared to aducanamab in MCI was also confirmed by another network meta-analysis (Terao et al. 2022).

Another set of data suggests that lithium could increase the survival of patients affected by ALS. Although the first trial of Fornai et al. (2008), was not followed by concordant experimental results (Aggarwal et al. 2010; Swash 2010), some recent trials suggest a potential therapeutic effect of lithium in combination with valproic acid (Boll et al. 2022). Interestingly, lithium levels in most trials were well beyond the threshold applied for maintenance in BD, ranging around 0.4–0.45 mEq/l. In addition, a dose-finding trial did not find a difference in the effectiveness of lithium at therapeutic (0.4–0.8 mEq/l) versus sub-therapeutic levels (0.2–0.4 mEq/L) (Chiò et al. 2010). Overall, even if lithium efficacy in ALS, especially in monotherapy, remains disputed, this would be exerted even at serum levels of lithium generally below the threshold considered effective in the maintenance of BD.

In sum, these studies suggest the effectiveness of subtherapeutic doses of lithium in modifying the detrimental trajectory of cognition in major cognitive disorders. Although the effectiveness of lithium monotherapy in ALS remains disputed, clinical effects have been observed at low doses.

### Biological effects of lithium at low concentrations: evidence from preclinical models

The mechanism through which lithium microdose may achieve this beneficial effect is still not known, but several studies have explored the hypothesis of modulation of molecular pathways involved in neuroprotection, including brain-derived neurotrophic factor (BDNF), glycogen synthase kinase 3 β, and inflammatory pathways. Most of the studies performed so far used lithium microdose formulations in rat models of AD, while, to the best of our knowledge, only one study used human-derived cell lines. The study by Nunes and co-workers (Nunes et al. 2015) used microdoses of lithium (0.25 mg/Kg/day in drinking water) to treat a transgenic mouse model of AD expressing a mutant form of the amyloid precursor protein. Findings showed improved spatial and aversive-related memory as well as a significant decrease in senile plaques, maintenance of neuronal density and increased BDNF density in cortex (Nunes et al. 2015). The same authors further explored this finding by evaluating the neuroprotective and anti-inflammatory effects of lithium in the organotypic hippocampal culture of a mouse model of accelerated ageing (SAMP-8). Findings showed that low doses of lithium (20 µl) were able to reduce the expression of genes coding for pro-inflammatory cytokines (IL-1α, IL-6) while increasing the expression of the anti-inflammatory cytokine IL-10 (Toricelli et al. 2021).

The involvement of BDNF in modulating the efficacy of lithium microdose was also tested in primary cultures of cortical and hippocampal neurons from rats (De-Paula et al. 2016). In this study, cell cultures were treated with different subtherapeutic (0.02 and 0.2 mM) and therapeutic (2 mM) concentrations of chronic lithium treatment (7 days). Interestingly, lithium 0.02 mM increased intracellular expression of BDNF in cortical and hippocampal neurons. Moreover, extracellular BDNF of cortical neurons increased by 30% and 428% at 0.02 and 0.2 mM, respectively, and hippocampal neurons increased by 44% at 0.02 mM. These findings strongly support a positive modulation of BDNF expression and protein levels even with subtherapeutic doses of lithium.

Another protein that has been suggested to modulate the efficacy of lithium microdoses is GSK-3β. GSK-3β is one of the first targets ever identified in the mechanism of action of lithium (Chatterjee and Beaulieu 2022), and there is robust evidence showing that GSK-3 inhibitors exert antidepressant-like effects (Chiu and Chuang 2010). GSK-3 is a multi-functional serine/threonine kinase widely expressed in the brain and linked to AD pathology (Cheng et al. 2024) as well as to psychiatric disorders (Manji et al. 1999). It is implicated in the modulation of several intracellular cascades, including insulin and neurotrophic signalling and modulation of the Wnt pathway, among others (Gould 2006). Through the interaction with these targets, GSK-3 regulates gene expression, embryonic development, neuronal survival, and circadian rhythms (Beurel et al. 2015). However, most of the in vitro studies exploring the effect of lithium on GSK-3 used doses that were equal to or higher than the therapeutic dose.

Zhao and coworkers conducted a study in which a novel formulation of lithium microdose (tri-lithium pyrroloquinoline quinone (Li3PQQ) was used to treat an animal model of AD (APP/PS1 trans-genic mice) (Zhao et al. 2014). Findings showed that Li3PQQ was able to restore memory and learning impairment and reduce cerebral amyloid deposition and phosphorylated tau levels. Interestingly, Li3PQQ inhibited the activity of GSK-3 and increased the activity of b-amyloid-binding alcohol dehydrogenase, which might underlie the beneficial effects of Li3PQQ on transgenic mice.

The study from Wilson and colleagues also supported that lithium micro-doses improve memory and learning, possibly through the interaction with GSK-3 (Wilson et al. 2017). In this study, the authors used a novel microdose formulation of lithium (NP03, formulation of 40 ug/Kg) to treat a rat model of AD (McGill-R-Thy1-APP) (Wilson et al. 2017). Notably, the treatment restored the memory loss of AD rats, stimulated hippocampal neurogenesis, reduced amyloid levels and rescued altered AD biomarkers. Specifically, GSK-3β, which was more active in AD rats compared to wild-type mice, was reversed by treatment with NP03. Moreover, lithium treatment determined a reduction in the expression of the beta-Secretase 1 gene (BACE1), which encodes the protein involved in the rate-limiting step in the Aβ production (Wilson et al. 2017).

Thus, Wilson’s and colleagues’ study is relevant in that it suggests that lithium doses far below the therapeutic level are capable of significantly inhibiting GSK-3β and that this mechanism could be involved in the observed improvements in memory and cognition.

Another recent study explored the role of GSK-3 in modulating the effects of low-dose lithium (Fenech et al. 2023). In this study, a mice model of AD (C57BL/6J) was fed with a low-dose lithium supplementation (10 mg/kg/day) for either 6 or 12 weeks. In the 12 weeks, arm mice were fed a chow diet, a high-fat diet, or a high-fat diet with lithium-supplemented drinking water. The results showed that GSK-3 activity was reduced in the PFC after 6 weeks of treatment. Moreover, in the 12-week study (which included an obese model), lithium supplementation reduced prefrontal cortex GSK-3 activity and improved insulin sensitivity.

Another study by Zhuo and coworkers explored the hypothesis that lithium can restore cognitive impairment through the stabilisation of abnormal Ca^2+^ activity in the brain of a murine model of schizophrenia (Zhuo et al. 2023). It has been proposed that Ca^2+^ activity can estimate alterations in the neural activity of brain regions. Moreover, accumulating evidence suggests that two-photon calcium imaging can associate altered Ca^2+^ activity in the prefrontal cortex (PFC) with cognitive or behavioural performance in a murine model of schizophrenia (C57BL/6) (Hamm et al. 2017, 2020; Yoon et al. 2022; Yuryev et al. 2018).

The study by Zhuo et al. visualised PFC Ca2 + activity and explored the effect of low doses of lithium on several parameters to characterise cognitive performance, including pre-pulse inhibition, novel object recognition, Morris water maze, and fear conditioning. The schizophrenia mouse model (MK801) showed cognitive impairment in all measures, as well as decreased Ca2 + activity. However, treatment with low doses of lithium (250 mg/d human equivalent dose) combined with quetiapine improved cognitive task at day 29 in all measures (Zhuo et al. 2023).

Finally, in a study by Schaeffer et al., the authors explored the effect of subtherapeutic doses of lithium (1.0 g lithium/kg chow) on restoring CA1 pyramidal neuron loss in a transgenic mouse model of AD (Schaeffer et al. 2017). Interestingly, lithium treatment at a low dose did not affect the CA1 neurons of the AD mice. In contrast, treatment of wild-type mice induced a significant increase in total CA1 pyramidal neuron number, which led to a significant increase in total CA1 pyramidal layer volume. These findings suggest that, while low-dose lithium can induce an increase in CA1 neuron number, intervention must be initiated as early as possible during neuropathological processes for beneficial effects to occur.

To the best of our knowledge, only one study has used human-derived cells to investigate the molecular targets of lithium microdose. Indeed, Viel et al. (2020) showed that microdose lithium treatment reduced cellular senescence and the senescence-associated secretory phenotype (SASP) in human astrocytes (Viel et al. 2020). SASP is characterised by the secretion of chemokines, cytokines, cell growth factors, and metalloproteases, leading to a tissue condition that may precipitate neurodegenerative processes. Thus, this study supports the hypothesis that lithium micro-doses may counteract neurodegenerative processes through modulation of inflammatory pathways. Overall, findings suggest that micro-doses of lithium interfere with intracellular targets that have also been implicated in studies using therapeutic doses.

## Conclusions

In this review, we have shown that lithium’s clinical and molecular effects are numerous and that its effects also appear to have a certain degree of specificity related to the dose.

In sum, the clinical effects of lithium are maximal for mood stabilization at concentrations higher than 0.6 mEq/l, although lower levels may be sufficient for preventing depressive recurrences in older populations of patients. A subset of individuals with BD may present an optimal response to lithium therapy even at relatively lower lithium concentrations. This subpopulation has been reported to represent nearly one-third of BD patients (Rybakowski 2014). Clinical features typically associated with a more positive lithium response have been delineated in the past and comprise a hyperthymic temperament, lack of cognitive impairment, a prominent episodic pattern, positive family history (Rybakowski 2014). In the era of personalized medicine, the possibility of further elucidating predictors of optimal lithium response and its eventual implementation in clinical practice represents certainly a tantalising prospect, albeit it is still far from potential clinical applications (Herrera-Rivero et al. 2023). How these elements may inform future research in identifying potential recipients of subtherapeutic lithium doses is unclear at this stage but may represent an additional area to explore. Certain lines of evidence suggest that lithium may exert an effect in reducing suicide risk. However, the rarity of this outcome and its dynamic nature between and within individuals virtually prevents the development and conduction of methodologically sound RCTs in this area of research, leaving only the possibility of using proxies for death by suicides such as suicide attempts. These may represent useful indicators of increased risk of death by suicide but may not necessarily aid in specifically identifying the population of individuals ultimately dying by suicide (Baldessarini and Tondo 2022; Pallaskorpi et al. 2017). The evidence for lithium efficacy in suicide prevention at subtherapeutic concentrations is also far from definitive, marred by the complexity of the study designs and shortcomings related to the limitations typical of observational reports.

Lithium’s ability to counteract cognitive decline appears to be exerted at subtherapeutic doses, possibly corresponding to its molecular neuroprotective effects. Evidence from preclinical and clinical models do not often overlap and point in the same direction, but this is certainly not true for lithium neuroprotective effects where both animal models of AD (Morris and Berk 2016; Nunes et al. 2015) and RCT results in AD patients appear to suggest a potential benefit of lithium therapy (Forlenza et al. 2011, 2019). Evidence from cohort and case-control studies in BD cognitive decline also seems to point in the same direction, finding a benefit for lithium therapy in reducing dementia risk by almost 50% in this population (Velosa et al. 2020). Indeed, lithium may reduce inflammation and induce neuroprotection even at doses several folds lower than those commonly used in the clinic to treat BD. Nevertheless, findings regarding its purported mechanism of action and supporting the hypothesized molecular underpinnings for lithium efficacy for this indication are missing, and most of the in vivo or in vitro studies relied on animal models of AD. Future studies should try to take advantage of patients-derived cellular models, such as neurons derived from induced pluripotent stem cells (iPSCs) or organoids, to better understand the mechanisms underlying the effect of lithium microdoses on brain health. Study selection was based on the varied expertise of the study authors, reflecting some of the most relevant evidence for this review’s subject. However, considering the narrative design for the present review, it is still possible that reports relevant to the discussed matters might have been omitted from the discussion.

## Data Availability

No datasets were generated or analysed during the current study.
